# Healthcare utilization in older patients using personal emergency response systems: an analysis of electronic health records and medical alert data: Brief Description: A Longitudinal Retrospective Analyses of healthcare utilization rates in older patients using Personal Emergency Response Systems from 2011 to 2015

**DOI:** 10.1186/s12913-017-2196-1

**Published:** 2017-04-18

**Authors:** Stephen Agboola, Sara Golas, Nils Fischer, Mariana Nikolova-Simons, Jorn op den Buijs, Linda Schertzer, Joseph Kvedar, Kamal Jethwani

**Affiliations:** 1 Partners Connected Health, Partner Healthcare, 25 New Chardon St., Suite 300, Boston, MA 02114 USA; 2 Massachusetts General Hospital, Boston, USA; 3 Harvard Medical School, Boston, USA; 4 Philips, Eindhoven, Netherlands

## Abstract

**Background:**

Personal Emergency Response Systems (PERS) are traditionally used as fall alert systems for older adults, a population that contributes an overwhelming proportion of healthcare costs in the United States. Previous studies focused mainly on qualitative evaluations of PERS without a longitudinal quantitative evaluation of healthcare utilization in users. To address this gap and better understand the needs of older patients on PERS, we analyzed longitudinal healthcare utilization trends in patients using PERS through the home care management service of a large healthcare organization.

**Methods:**

Retrospective, longitudinal analyses of healthcare and PERS utilization records of older patients over a 5-years period from 2011–2015. The primary outcome was to characterize the healthcare utilization of PERS patients. This outcome was assessed by 30-, 90-, and 180-day readmission rates, frequency of principal admitting diagnoses, and prevalence of conditions leading to potentially avoidable admissions based on Centers for Medicare and Medicaid Services classification criteria.

**Results:**

The overall 30-day readmission rate was 14.2%, 90-days readmission rate was 34.4%, and 180-days readmission rate was 42.2%. While 30-day readmission rates did not increase significantly (*p* = 0.16) over the study period, 90-days (*p* = 0.03) and 180-days (*p* = 0.04) readmission rates did increase significantly. The top 5 most frequent principal diagnoses for inpatient admissions included congestive heart failure (5.7%), chronic obstructive pulmonary disease (4.6%), dysrhythmias (4.3%), septicemia (4.1%), and pneumonia (4.1%). Additionally, 21% of all admissions were due to conditions leading to potentially avoidable admissions in either institutional or non-institutional settings (16% in institutional settings only).

**Conclusions:**

Chronic medical conditions account for the majority of healthcare utilization in older patients using PERS. Results suggest that PERS data combined with electronic medical records data can provide useful insights that can be used to improve health outcomes in older patients.

## Background

In most developing and developed countries, older adults are increasingly becoming the largest age bracket due to increasing longevity and lower fertility rates [1]. In the United States (US), it is estimated that between 2012 and 2050 the proportion of individuals 65 years and older will nearly double [2]. Advances in healthcare have succeeded in helping people live longer, but the gains have not translated to healthier lives in later life. Studies suggest that as people age, the risk of chronic diseases increases, leading to higher healthcare spending in later years [2]. Rising health expenditures of older adults put increasing fiscal pressure on Medicare, a federal health insurance program for US citizens who are 65 years and older, younger people with certain disabilities, and those who suffer from end stage renal disease [3]. In 2015, for example, total Medicare expenditure was $632 billion; approximately 25% of this cost was due to in-hospital care [4]. An additional 10% of the total cost was due to readmissions through emergency transports alone, which are common in older adults [5].

Notably, nearly a quarter of hospital readmissions in the Medicare cohort are considered potentially avoidable [6]. The definition of avoidable readmissions varies depending on patient demographics, available resources, settings, and pre-existing conditions [6]. To incentivize hospitals to reduce potentially avoidable readmissions, Centers for Medicare and Medicaid Services (CMS), the federal agency that administers Medicare, implemented initial policies in 2012 to penalize hospitals with above-average 30-days readmissions for heart failure, pneumonia, and acute myocardial infarction. In 2014, the applicable conditions were expanded to include patients admitted for acute exacerbation of Chronic Obstructive Pulmonary Disease (COPD), elective total hip arthroplasty and total knee arthroplasty. Consequently, this policy change motivated hospitals to implement a variety of quality improvement programs and technological innovations to improve outcomes [7]. So far, results are encouraging. For example, in 2013, CMS reported a reduction in readmission rates from an average of 19.0% from 2007–2011 to 17.5% in 2013 [8]. Moreover, while CMS has traditionally reimbursed conditions individually for a single illness or a course of treatment, the Bundled Payments for Care Improvement Initiative scheduled for 2017 is designed to help healthcare providers better manage those with multiple chronic conditions, mental and behavioral health issues, cognitive impairment and mobility-related disabilities.

Gerontechnologies are commonly used to support and improve outcomes in older individuals. Their use is supported by studies showing that as people age, they want to continue living independently at home for as long as possible [9, 10]. Personal emergency response systems (PERS), one group of such technologies, are traditionally used as fall alert systems. Generally, PERS consist of 3 components: 1) a help push button that is worn either as a necklace or a bracelet 24 h a day, 2) an in-home communication system and 3) an emergency response center. Patients may press the help button at any time to activate the in-home communication system that connects to the response center. The response center associate inquires about the situation and contacts either an informal responder (e.g. neighbor, a family member) or an Emergency Medical Service (e.g. ambulance, police, or fire department) based on the patient’s specific situation, and then follows up to confirm that help has arrived.

PERS are used worldwide. Previous studies reported that they enhance quality of life by reducing anxiety about falling and improving confidence in performing everyday activities [11]. The system also provides older adults with a sense of security knowing that they can quickly get help in an emergency [12–14]. Additionally, a majority of users generally find it acceptable and easy to use, with little or no need for extensive training, and are satisfied with the system [14]. Although PERS is generally well-accepted, some studies have identified end user experiences related to resistance and non-use of the technology [14–16]. Lack of technological skills accounted for some of the reasons for not engaging with PERS. Non-technological explanations for not engaging with the device included having other options for safety, not wanting to be bothered or to be a burden to others, or a perceived stigma of using PERS [14, 17]. While these studies evaluated users’ perspective for usefulness, acceptability, satisfaction and other related qualitative measures, there remains a dearth of high quality quantitative evaluation of healthcare utilization in patients using PERS. Therefore, in this study we conducted a retrospective analysis to evaluate healthcare utilization rates over a 5-year period in a population of older patients using PERS purchased through the home care service of a large healthcare organization.

## Methods

### Aims

The primary aim of this study was to evaluate healthcare utilization of PERS patients as measured by 30-, 90- and 180-day readmission rates. As secondary objectives, we evaluated the principal admitting diagnoses, discharge dispositions, and the prevalence of medical conditions leading to potentially avoidable admissions. Additionally, we analyzed PERS help button pushes in terms of type of incidents and prevalence of emergency transport.

### Design

This is a retrospective longitudinal medical record review and analyses to evaluate hospital utilization in older patients using PERS. The study was approved by the Partners Human Research Committee, the Institutional Review Board for Partners Healthcare hospitals.

### Settings

Patients included in this study were residents of the Greater Boston Area who used PERS and received care through Partners HealthCare at Home (PHH) for any length of time between 2011 and 2015. PHH is a home care management service that offers general care as well as specialized services to help patients manage chronic conditions at home. PHH serves all patients within the Partners Healthcare System, a private, nonprofit network of seven major hospitals, including two large academic centers and several community health centers. Patients spend, on average, 2–3 months in the home care service; however, participants may have varying care duration due to individualized care for each patient, and a patient may be enrolled into PHH more than once (i.e. they may be re-enrolled after a prior discharge from PHH service). In addition to in-person home visits, PHH utilizes a variety of technological innovations to remotely monitor patients and deliver high quality clinical care. One of these technologies is the Philips Lifeline’s PERS, which is routinely recommended for older adults or chronically ill patients who are at risk of falls or other health-related emergencies at program enrollment. While patients are usually referred to the PERS service by their care provider, some patients came to use the PERS system through external referrals directly from the market or from a family member’s recommendation. Unlike some countries (e.g. Australia) where long-term care insurance policies and health maintenance organizations are used to subsidize or fully reimburse for PERS [18], it is typical in the US for PERS to be obtained through private payment. Thus in this study, the PERS service was privately paid for by patients and not covered by their health insurance. A majority of patients continued to use PERS even after discharge from home care.

### Data sources

The primary data sources for this study were the Enterprise Data Warehouse (EDW) and Research Patient Data Registry (RPDR) — both are electronic medical record data repositories of hospitals within the Partners Healthcare System network, which include data such as demographic information, diagnoses, hospital utilization and healthcare costs. CMS 2014 population comparison data were collected from “2014 CMS Statistics - Centers for Medicare & Medicaid Services “available at www.cms.gov [19]. The primary PERS data source for this project was the Philips Lifeline database, which includes the following historical data: demographics, patients’ living situation and care giver network, self-reported medical conditions, and medical alert data. Medical alert data includes all information gathered during the interactions of the patients with the Lifeline response center associates, including the reason the personal help button was pressed and the outcome of the patient-response center interaction.

### Subject selection

Patients were selected for inclusion in this study through a cross-reference of Philips Lifeline and Partners Healthcare at Home (PLL/PHH) patients. 4290 PERS users at PHH were identified from the Philips’ database and were cross-matched to the Partners Healthcare data warehouse. Identified patients were matched by first and last names and date of birth and/or address. We extracted 5 years of healthcare utilization data (2011–2015) for all identified patients. After matching and data extraction, patients were assigned de-identified study IDs and the data was stripped of all personal identifiers before analyses. We excluded unmatched patients or matched patients without utilization record in the data warehouse.

### Outcomes

The primary outcome was to characterize the healthcare utilization of PERS patients. This outcome was assessed by 30-, 90-, and 180-day readmission rates using clinical encounter data in the EDW. Index admissions are used as a reference point for subsequent admissions. The number of days from an index admission to the next admission is used to determine the type of readmission, 30-, 90-, or 180-day.

Additionally, we assessed the frequency of principal admitting diagnoses. This was determined by analyzing the principal diagnosis International Classification of Diseases (ICD)-9 code associated with each inpatient admission, then categorizing the codes according to the condition groups designated by the Health Cost and Utilization Project (HCUP) Clinical Classifications Software (CCS), which characterizes diagnoses into clinically meaningful groups making it easier to see diagnosis data patterns [20].

We also evaluated the prevalence of medical conditions leading to potentially avoidable admissions. This was determined by analyzing the principal diagnosis ICD9 code associated with each inpatient admission and categorizing the codes according to 3 groups: those potentially avoidable in both institutional and non-institutional settings, those potentially avoidable only in institutional settings [6, 21], and those which are not determined to be potentially avoidable.

Finally, PERS utilization data was summarized. Each personal help button press was registered in the PERS database as a unique “case”. Case data included date of the press, the case type, the situation involved, and the outcome of the press.

### Statistical plan

Demographic and healthcare utilization data for the fiscal years 2011–2015 were extracted from the EDW using Structured Query Language (SQL) and Microsoft SQL Server Management Studio (SSMS) 2014. Data management and de-identification were achieved through SSMS and Microsoft Excel 2007. Descriptive analyses were completed with Excel, Tableau Software 9.2. R was used for linear regression analyses using RStudio version 3.2.2. Pearson chi-squared tests were used for categorical variables and t-tests for continuous variables using STATA version 14.1.

## Results

### Population identification

**Fig. 1:**
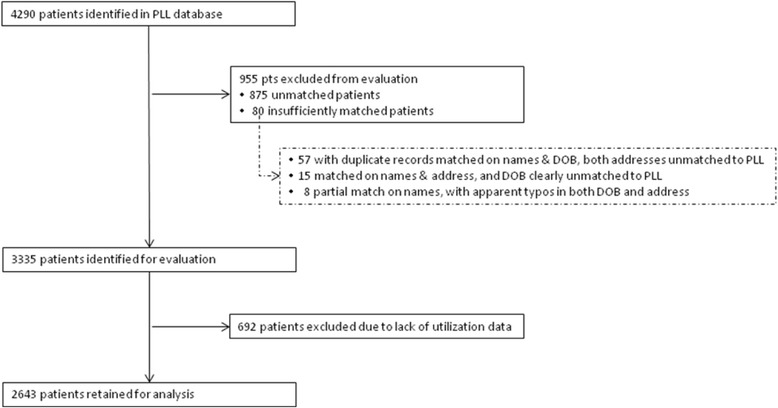
Sample Selection Process. Legend: outlines the sample selection process. 78% (3335/4290) of patients on the list of PLL enrollees were identified from the EDW; and 79% (2643/3335) of those identified met the criteria for inclusion in the study

“Figure 1. Sample Selection Process” details the sample selection process. 78% (3335/4290) of patients on the list of PLL enrollees were identified from the EDW; and 79% (2643/3335) of those identified met the criteria for inclusion in the study.

### Demographics

The study population was, on average, significantly older than the national CMS population (79 years old vs. 71 years old). Majority (75%) of the study population was female in comparison to the younger CMS population, where 55% of all beneficiaries were female. Similar to national CMS population estimates, a majority (93%) of the study population identified as white (Table 1). It should be noted that there was a large proportion of unknown values for education and marital status, therefore it is difficult to make conclusive comparisons between the two populations based on these data. The percentages presented in Table 1 exclude the unknown values.

**Table 1:** Subject Demographic Characteristics

Variable	PLL/PHH	2014 CMS (A + B) national	*p-value*
	% (*N* = 2,643)	% (*N* = 51,470,589)	
Age			
Mean, (SD)	79 (11)	71.1	<0.001
Age Cat.			<0.001
< 65	11 (303)	16 (8,352,750)	
65+	89 (2340)	84 (43,117,839)	
Gender			<0.001
Female	75 (1,990)	55 (28,197,507)	
Race			
White	93 (2312)	80 (41,269,942)	0.41
Hispanic	0 (9)	6 (2,975,486)	<0.001
Black/AA	5 (128)	10 (4,877,000)	<0.001
Other	1 (26)	5 (2,348,162)	<0.001
*Unknown*	*n = 168*	*NA*	*NA*
Education			
≥ College	36 (551)	27 (13,979,412)	<0.001
Some college	7 (102)	25 (12,821,324)	<0.001
High-school	43 (657)	28 (14,561,030)	<0.001
< High-school	13 (201)	20 (10,103,677)	<0.001
*Unknown*	*n = 1132*	*NA*	*NA*
Marital Status			
Married	29 (695)	52 (26,672,059)	<0.001
Divorced	13 (317)	17 (8,605,883)	<0.001
Single	20 (475)	9 (4,658,088)	<0.001
Widowed	37 (887)	22 (11,539,706)	<0.001
*Unknown*	*n = 269*	*NA*	*NA*

### PERS utilization data

Of the 2643 patients included in the analyses, 97% (*n* = 2555) utilized PERS for a median of 3 years (interquartile range, 1–5). 28.4% of patients have button utilization data for all 5 years of the evaluation period, 22.3% between 3–4 years of button utilization data, and 49.4% have 1–2 years of button utilization data. Overall, there were 78,585 registered cases. Case types included regular maintenance tests of PERS (68.7%), other maintenance or servicing of the system (13.9%), accidental button presses (11.7%), and adverse ‘incidents’ (5.4%). Less than 1% (0.3%) of cases were of the type ‘other’ or unknown.

Of all incident cases (*n* = 4231), situations described as falls accounted for 43.2%, while physical and psychological symptoms (such as breathing, pain, bleeding/injury, illness, dizziness, confusion and anxiousness) accounted for 42.7%. Another 6.5% of incident cases were for various personnel requests which included general, responder, transportation, nursing, medication and accessory requests; and 2.0% were due to a variety of issues including equipment issues, adverse external situations (utility problems, domestic problems, fire, or being locked out of the home) or benign situations (accidental alarms, patients asking for the time, or patients wanting somebody to talk to). The remaining 5.6% of incident cases were captured in unstructured text pertaining to topics not included in any of the listed categories. Further analysis of incident data revealed that 70% of incident cases with physical/psychological symptoms resulted in an emergency transportation compared with 29% of fall-related incidences that resulted in transportation (risk ratio = 2.41; *p* < 0.001). For other categories of incident cases, 25% and 28% of personnel and other forms requests resulted in transportation respectively.

### 30-, 90-, and 180-day readmissions

The study cohort had a total of 123,753 inpatient and outpatient encounters over the 5-year evaluation period. 66% (1734/2643) of patients had inpatient admissions, with a total of 5258 admissions, accounting for 4% of the total inpatient and outpatient encounters. Over the 5 year period, inpatient admissions generally increased from 3.5 to 5.7% in proportion to outpatient encounters (Table 2).

**Table 2:** Clinical Encounter details, 2011–2015

	2011	2012	2013	2014	2015	Overall
Total	*n* = 22,479	*n* = 24,045	*n* = 25,262	*n* = 26,900	*n* = 25,067	*N* = 123,753
Outpatient encounters, % of Total (n)	96.5 (21,696)	96.2 (23,121)	96.2 (24,298)	95.7 (25,740)	94.3 (23,640)	95.8 (118,495)
Inpatient admissions, % of Total (n)	3.5 (783)	3.8 (924)	3.8 (964)	4.3 (1,160)	5.7 (1,427)	4.2 (5,258)
Readmission Rates						
30-days	14.3	11.9	15.0	17.2	16.4	14.2
90-days	27.7	27.8	32.7	35.2	34.5	34.4
180-days	38.3	39.2	43.5	44.2	43.9	42.2

**Fig. 2:**
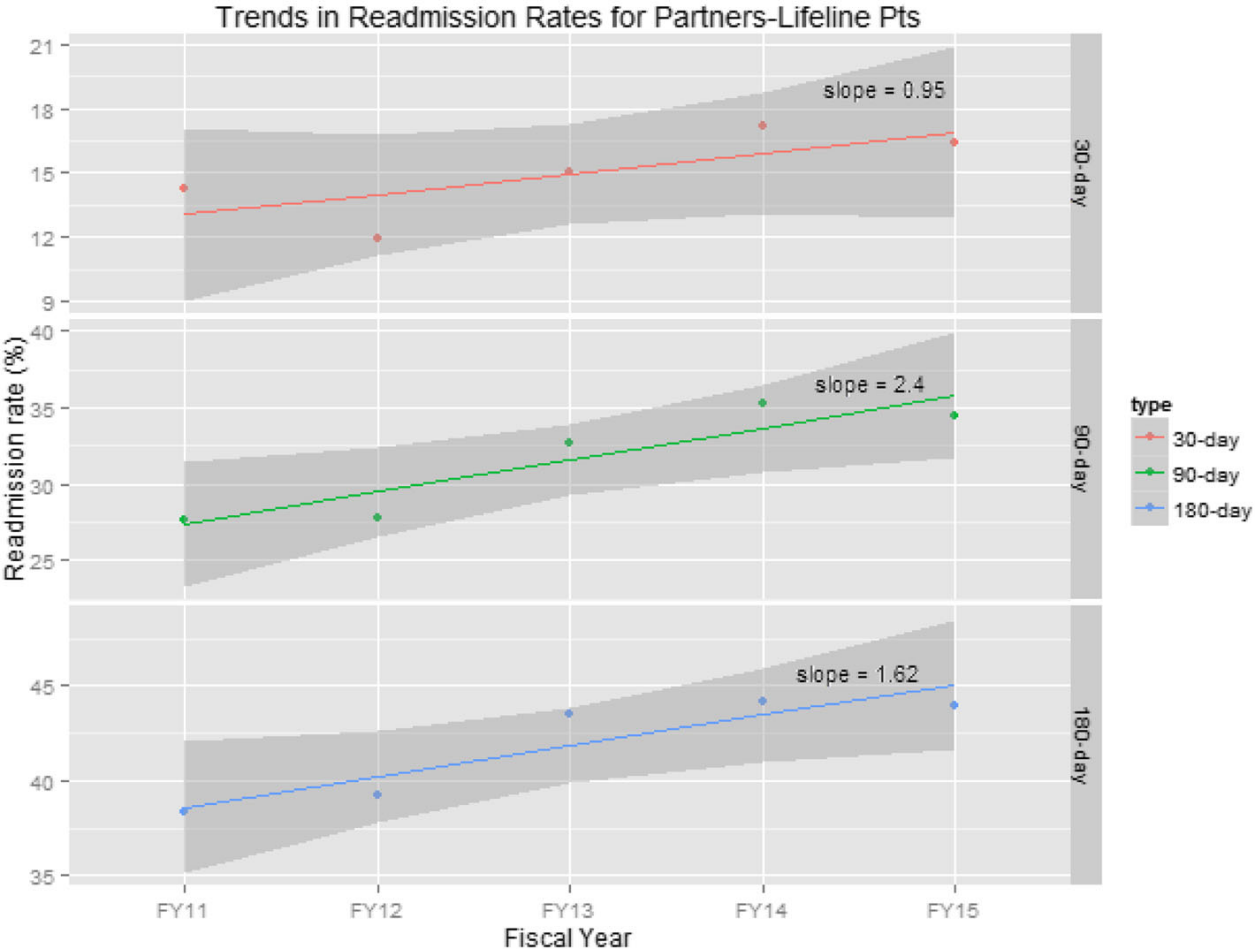
Patient 30-, 90-, and 180-day Readmission Rates from FY11 to FY15. Legend: illustrates the rates of 30-, 90-, and 180-day readmissions from FY11 to FY15. While 90- and 180-day readmissions increased significantly (*p* = 0.03 and *p* = 0.04, respectively), the increase of 30-day readmissions was not statistically significant (*p* = 0.16)

The overall 30-, 90-, and 180-day readmission rates were 14.2, 34.4, and 42.2%, respectively (Table 2). The rates gradually increased from 2011 to 2015 (Fig. 2. Patient 30-, 90-, and 180-day Readmission Rates from FY11-FY15). While 30-day readmission rates did not increase significantly (*p* = 0.16), 90- and 180-day readmission rates did increase significantly (*p* = 0.03 and *p* = 0.04, respectively).

### Discharge disposition

**Fig. 3:**
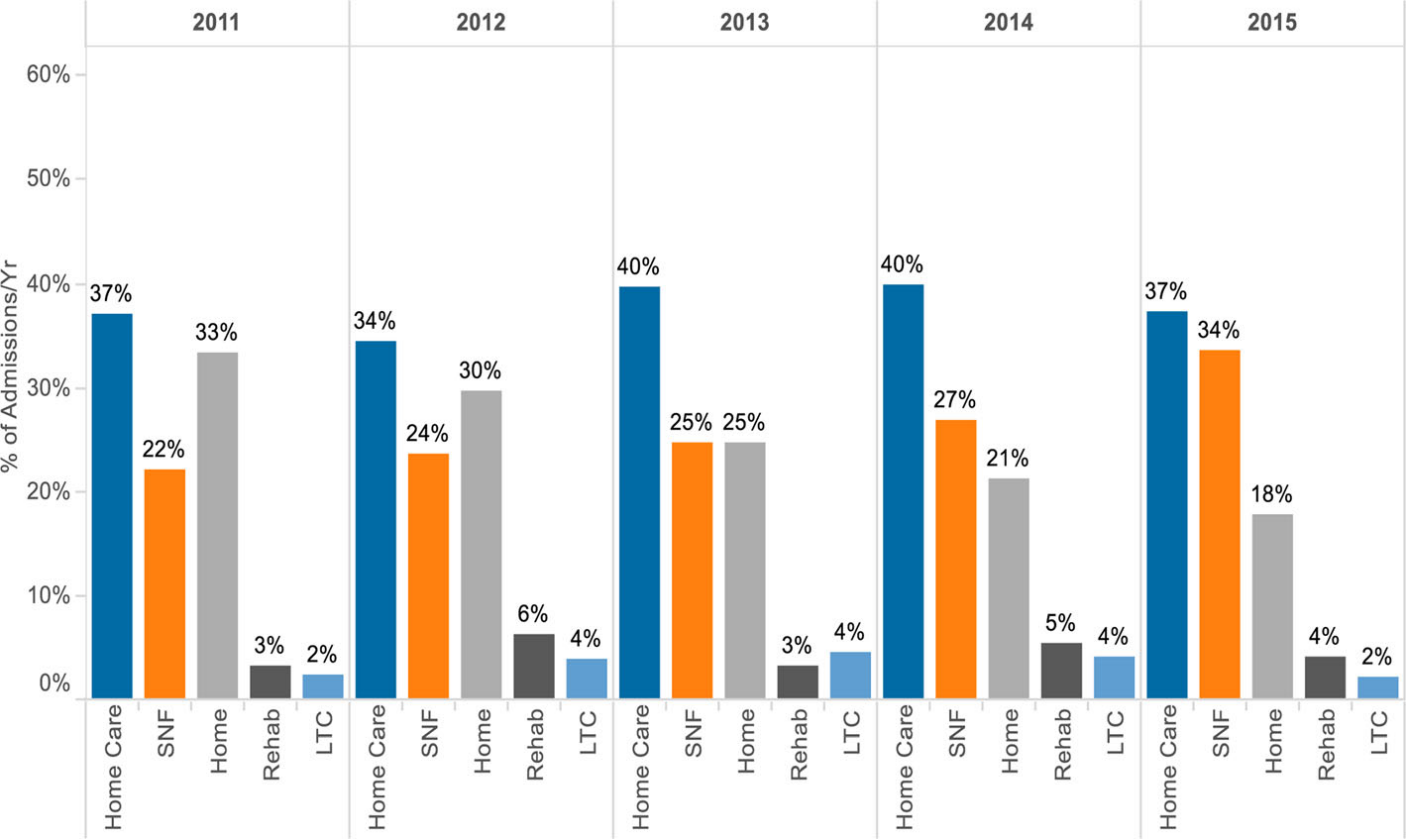
Discharge Dispositions by Year (%). Legend: details discharge dispositions from FY11 to FY15. In all 5 years, the most frequent discharge dispositions from inpatient admissions went to home care. Notably, from FY11-FY15, those discharged home generally decreased from 33 to 18% while those discharged to SNFs increased from 22 to 34%

As expected, given the study population was known to use home care, the three most frequent discharge dispositions from inpatient admissions were to home care, a skilled nursing facility (SNF), and home. Notably, from 2011–2015, those discharged home generally decreased from 33 to 18% while those discharged to SNFs increased from 22 to 34% (Fig. 3. Discharge Dispositions by Year, %).

### Potentially avoidable admissions

Overall, 63% of admissions were not associated with principal diagnoses coded as potentially avoidable; 21% of admissions were associated with principal diagnoses coded as potentially avoidable in either an institutional or non-institutional setting; and 16% were associated with principal diagnoses coded as potentially avoidable in an institutional setting only.

The most frequent all-setting conditions associated with potentially avoidable admissions in this population were congestive heart failure (CHF) (6% of all admissions), followed by COPD (5%) and urinary tract infections (4%). For conditions avoidable in institution-only settings, falls and trauma were most frequent at 6.5%, followed by pneumonia (5%) and skin ulcers/cellulitis(2%) (Table 3).

**Table 3:** Percentage (%) of admissions with Principal Diagnoses falling under CMS-defined Potentially Avoidable Conditions

	2011	2012	2013	2014	2015	Overall
Number of admissions	*n* = 783	*n* = 924	*n* = 964	*n* =1160	*n* = 1427	*N* = 5258
Non-avoidable	65.9	66.3	64.8	60.5	60.2	63.0
Potentially avoidable*	34.1	33.7	35.2	39.5	39.8	37.0
* Institutional and non-institutional settings + institutional settings only conditions						
*Conditions for both institutional and non-institutional settings*						
Overall	18.1	17.9	21.8	24.0	22.1	21.1
Congestive heart failure	5.0	4.4	5.8	6.7	7.4	6.1
COPD, chronic bronchitis, and asthma	4.3	4.9	6.8	6.6	4.3	5.4
Urinary tract infection	3.6	2.7	3.1	3.6	4.8	3.7
Dehydration, volume depletion including acute renal failure and hyponatremia	2.6	3.6	3.2	4.1	2.5	3.2
Hypertension and hypotension	1.3	1.3	1.0	1.7	1.1	1.3
Seizures	0.8	0.9	1.2	0.7	0.6	0.8
Poor glycemic control	0.3	0	0.1	0.1	0.9	0.3
Constipation, fecal impaction, and obstipation	0.1	0.1	0.3	0.2	0.1	0.2
Weight loss (failure to thrive) and nutritional deficiencies	0.3	0	0.1	0.3	0.4	0.2
*Conditions for institutional settings only*						
Overall	16.0	15.8	13.4	15.5	17.7	15.8
Falls and trauma	6.0	6.8	4.4	6.6	7.9	6.5
Pneumonia and bronchitis (lower respiratory illness)	4.6	4.5	4.4	5.0	4.8	4.7
Skin ulcers and cellulitis	2.6	2.1	2.1	1.7	1.8	2.0
Diarrhea, gastroenteritis, and C.difficile	2.2	1.6	2.1	1.4	2.2	1.9
Anemia	0.3	0.5	0.5	0.7	0.4	0.5
Psychosis, severe agitation, and organic brain syndrome	0.3	0.1	0	0	0.4	0.2
Altered mental status, acute confusion, and delirium	0.1	0.1	0	0.2	0.2	0.1

* Institutional and non-institutional settings + institutional settings only conditions

### Principal inpatient diagnoses

The top 10 principal admitting diagnoses included CHF (5.7%), COPD (4.6%), dysrhythmias (4.3%), septicemia (4.1%), pneumonia (4.1%), urinary tract infection (3.7%), device complications (3%), osteoarthritis (3%), acute cerebrovascular disease (3%) and hip fracture (2.8%) (Table 4).

**Table 4:** Top 10 Inpatient Principal Diagnosis Groups, %

	2011	2012	2013	2014	2015	Overall
% of inpatient admissions	*n* = 783	*n* = 924	*n* = 964	*n* = 1160	*n* = 1427	*N* = 5258
CHF (non-hypertensive)	4.9	4.3	5.5	6.2	6.9	5.7
COPD	3.8	4.5	5.9	5.7	3.4	4.6
Dysrhythmias	5.0	5.2	4.4	3.6	3.7	4.3
Septicemia	1.9	3.0	4.8	4.2	5.4	4.1
Pneumonia	4.0	4.2	4.1	4.1	3.9	4.1
Urinary tract infection	3.6	2.7	3.1	3.6	4.8	3.7
Device complications	3.7	3.5	2.6	2.8	2.9	3.0
Osteoarthritis	4.0	3.7	2.7	2.4	2.8	3.0
Acute cerebrovascular disease	2.8	2.8	2.0	4.7	2.5	3.0
Hip fracture	2.3	3.2	1.3	3.0	3.7	2.8

## Discussion

To the best of our knowledge, this is the first study to evaluate healthcare and PERS utilizations in a large cohort of patients. Our analyses revealed some key findings. First, over the 5-year study period, hospitalizations increased progressively. In particular, we observed significant increases in 90- and 180-day, but not 30-day, readmission rates. Second, patient referrals for further care (home health care and skilled nursing care) after hospital discharge increased progressively, with skilled nursing care referrals accounting for the increase. In contrast, the proportion of patients discharged home decreased. Third, chronic medical conditions were the most frequent principal admitting diagnoses. Fourth, 37% of all admissions qualified as potentially avoidable admissions in institutional and non-institutional settings combined. Fifth, the PERS help button was mainly used to access help for acute or chronic condition- related symptoms and fall-related events; but significantly more symptom-related cases resulted in emergency room transport compared with fall-related events.

Older patients with chronic diseases represent an increasingly expensive segment of the population due to higher hospital utilization rates. Healthcare systems in the era of the Affordable Care Act (ACA) – a health reform law in the US that includes a series of mandates, subsidies, and insurance exchanges designed to control rising healthcare costs and increase insurance coverage [22] – are financially incentivized to maintain 30-day readmissions below the national average [23]. Over the 5-year period, this study showed that 30-day readmissions were relatively steady whereas 90- and 180-day readmissions increased significantly, along with the number of overall admissions. This finding is similar to a previous study which showed that 30-day readmission rates at the national level fell sharply as a result of the ACA [24]. Still, evidence is inconclusive regarding how 90- and 180-day readmission rates were affected by patient management strategies implemented by accountable care organizations to reduce readmissions [24]. Under recently proposed bundled payment models, hospitals admitting patients with applicable conditions are accountable for the cost and quality of care provided to Medicare fee-for-service beneficiaries during the inpatient stay and in the 90-day period post-discharge. These policies promote opportunities for hospitals to better coordinate care among healthcare providers, support risk assessment and avoid complications with the goal of improving the overall quality of care and decreasing cost.

In an analysis of the most frequent hospital conditions in 2011 reported by Healthcare Cost and Utilization Project, CHF, pneumonia, septicemia, and cardiac dysrhythmias were in the top 5 most frequent conditions among adults ages 65–84 and age 85 and older [25]. This is similar to our study findings. Furthermore, our finding that a fifth of all admissions were potentially avoidable is similar to recent national estimates in Medicare beneficiaries [6]. This, therefore, presents an opportunity to implement population health management strategies to decrease disease complications and hospitalization rates [26, 27]. For example, in a telemonitoring program for CHF patients at the Massachusetts General Hospital, results showed significant decreases in 30-day readmission rates and overall hospitalization rates over the 4-month program period [7]. Expanding such programs and complementing them with risk assessment algorithms to target the interventions to the most high-risk patients will yield the most cost-efficient impact.

While PERS are traditionally used as fall alert systems, our findings that the most frequent principal admitting diagnoses were not related to fractures but rather due to conditions associated with potentially avoidable admissions, and that most emergency transportations were due to symptom-related events rather than falls, demonstrates that PERS can be equally useful in chronic disease management programs. PERS complements most integrative care systems by facilitating rapid medical response when a patient engages the personal help button. One study showed that, 1 year after receiving a PERS unit, patients experienced decreased levels of fear, stress and anxiety about their well-being [28]. These psychological effects can contribute to improved self-efficacy for self-care and improve clinical outcomes in chronic diseases management. However, in certain contexts and population groups, there are possibilities of unforeseen consequences of using PERS, such as decreased interactions with family members [11].

This study has some limitations. First, as shown in Table 1, the study population is older, more female, more educated, more likely to be single or widowed, and more likely to identify as white in comparison to the general Medicare population. In addition, the PERS service used in this population is privately paid for by patients and not covered by their health insurance. This may limit the generalizability of the study to educated, older women that can afford the service. Also, our analyses exclude clinical encounters not captured in the medical records or in the claims reported in the Partners Healthcare system.

## Conclusions

Support for chronic conditions is a high priority in caring for older patients using PERS. Interventions to manage conditions causing potentially avoidable admissions are needed. PERS data combined with EMR data can provide useful insights to improve our understanding of chronic disease management. Future studies evaluating patterns in medical alert data from the button presses, combined with data from other remote monitoring technologies and medical records data, may be useful in predicting hospitalizations and improving the quality of care. Additionally, future studies should focus on demonstrating the cost implications of healthcare utilization data in PERS users and the potential role of PERS in the delivery of value-based care.

## Abbreviations

CCS: Clinical classifications software
CHF: Congestive heart failure
CMS: Centers for medicare and medicaid services
COPD: Chronic obstructive pulmonary disease
EDW: Enterprise data warehouse
HCUP: Healthcare cost and utilization project
ICD: International classification of disease
PERS: Personal emergency response system
PHH: Partners healthcare at home
PLL: Philips lifeline
PLL/PHH: Philips lifeline and partners healthcare at home
RPDR: Research patient data registry
SNFs: Skilled nursing facilities
SSMS: Microsoft SQL server management studio
